# Tracheobronchopathia osteochondroplastica: a case report highlighting the importance of clinico-radiologic correlation

**DOI:** 10.1259/bjrcr.20230062

**Published:** 2023-10-24

**Authors:** Naomi Jillan V. Rodriguez, Jeffrey T. Manto, Paula Marie M. Sydiongco-Inocencio, Janine Marriah G. Dela Cruz, Gabriel Martin S. Ilustre, Anna Pamela C. Dela Cruz

**Affiliations:** 1 Department of Radiology, University of the Philippines–Philippine General Hospital, Manila, Philippines; 2 Department of Otolaryngology-Head and Neck Surgery, University of the Philippines–Philippine General Hospital, Manila, Philippines

## Abstract

Tracheobronchopathia osteochondroplastica (TBPO) is a rare and benign idiopathic disease of the tracheobronchial tree, characterized by osseous, and/or cartilaginous submucosal nodules involving the anterior and lateral walls of the airways with sparing of the posterior wall. We present a case of a 51-year-old non-smoker female, presenting with a 2-year history of gradually enlarging anterior neck mass with foreign body sensation, frequent throat clearing, and occasional hoarseness. She was initially diagnosed with recurrent respiratory papillomatosis due to the presence of nodules on flexible laryngoscopy. A plain neck and chest CT then showed irregularity of the tracheal walls with calcified nodules projecting into the lumen, sparing the posterior wall, consistent with TBPO. Fiberoptic bronchoscopy with biopsy was also done which confirmed the inferior extent of the nodules down to the level of the carina, and the presence of fragments of mature bone tissue within the nodules.

## Case presentation

We report a case of a 51-year-old non-smoker female who had a 2-year history of a gradually enlarging anterior neck mass, with associated globus sensation, frequent throat clearing, and occasional hoarseness. Due to the persistence of symptoms, she consulted at our institution for evaluation and management. On physical examination, she had a palpable anterior neck mass that moves with deglutition.

## Investigations

**Figure 1.**
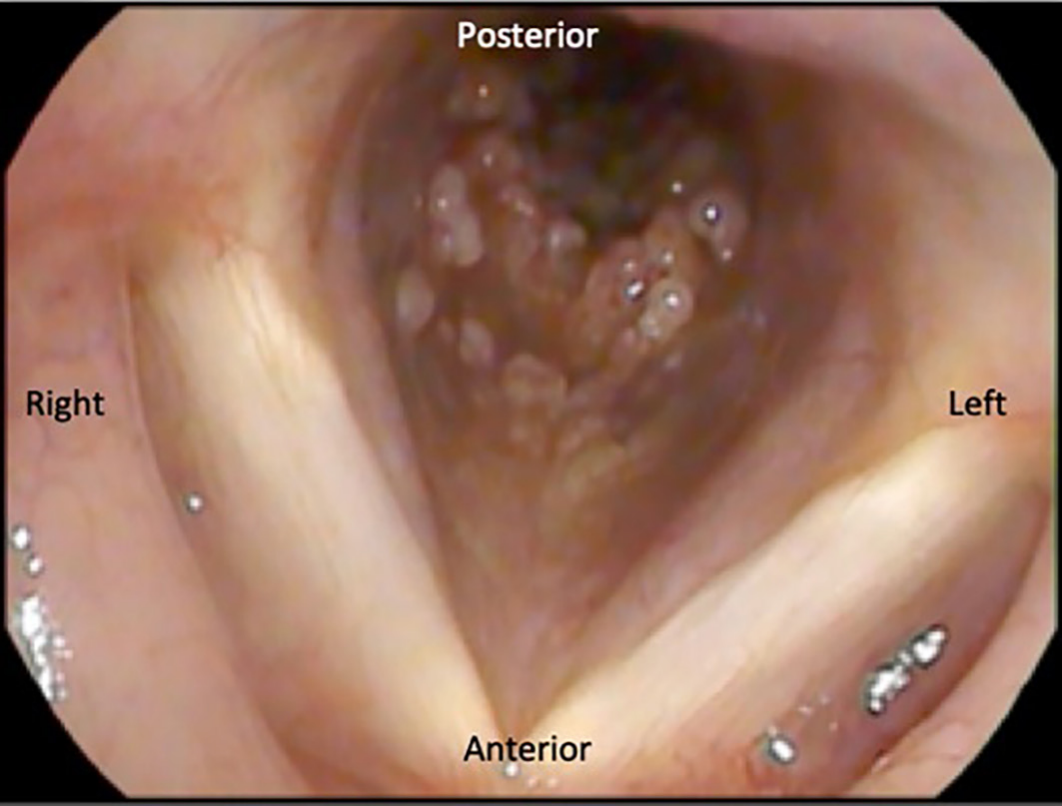
Flexible laryngoscopy. Multiple papillomatous lesions in the anterolateral wall of the trachea at the level of the subglottis.

Flexible laryngoscopy was done and incidentally, multiple lesions were seen in the subglottic area. These lesions appeared to be wart-like and were seen in the anterolateral wall of the subglottis and visualized portion of the trachea, sparing the supraglottis and glottis (Figure 1). The patient was initially diagnosed with recurrent respiratory papillomatosis.

**Figure 2.**
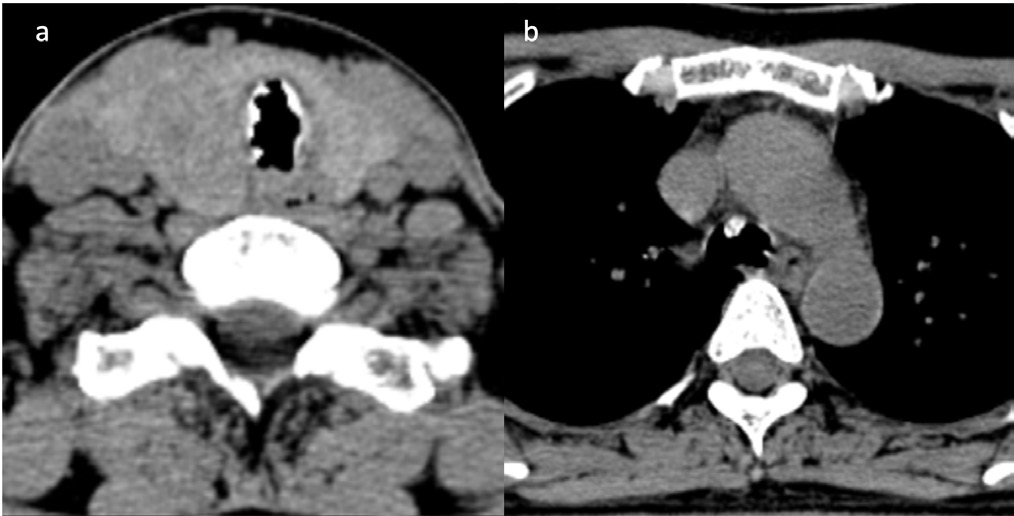
Axial unenhanced neck and chest CT scan at the level of the thyroid gland (**a**) and mediastinum (**b**). Irregularity of the tracheal walls with calcified nodules projecting into the lumen, sparing the posterior wall and extending down to the carina.

**Figure 3.**
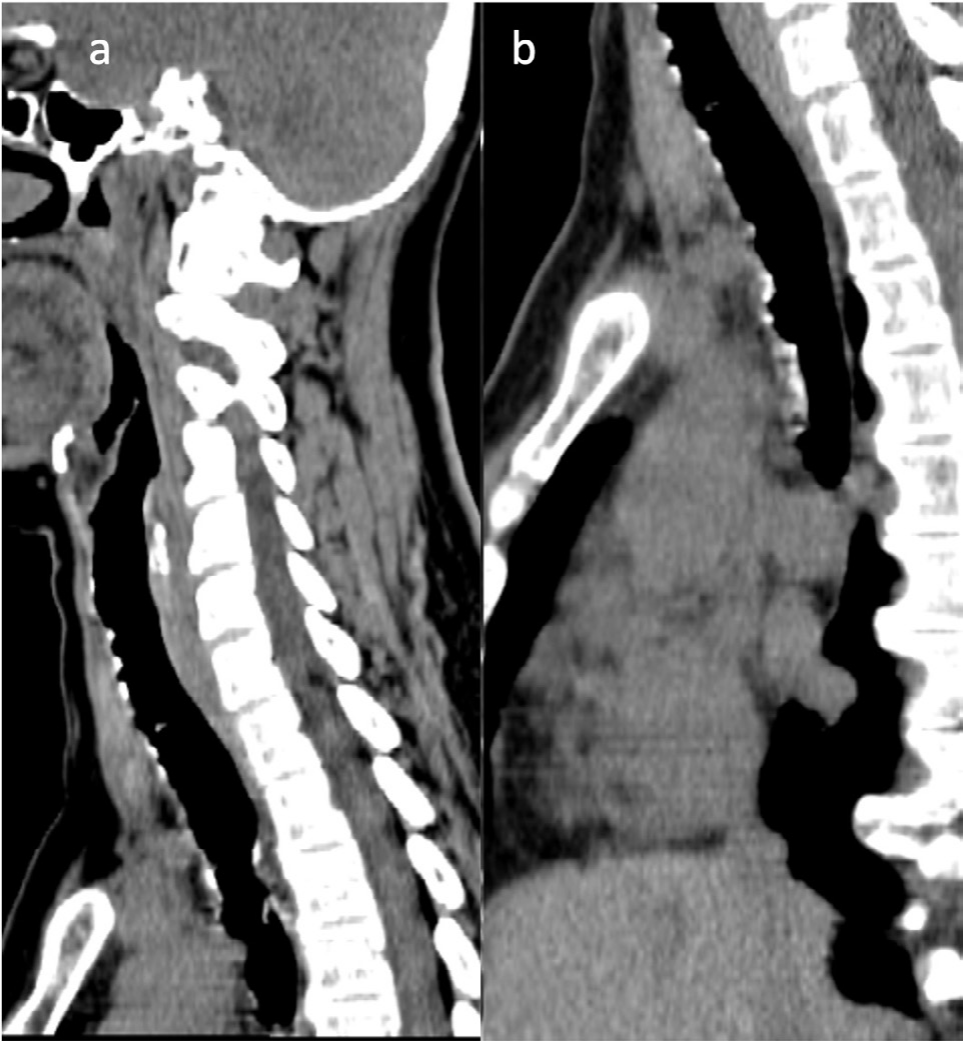
Sagittal unenhanced neck and chest CT scan showing the upper (**a**) and lower trachea (**b**) which exhibit irregularities of the anterior wall, sparing the posterior wall.

A plain neck and chest computed tomography (CT) scan was done for further evaluation of the subglottic lesions and to determine its extent. The imaging study revealed the classic findings of irregularity of the tracheal walls, with calcified nodules projecting into the lumen, sparing the posterior wall (Figure 2). The lesions spanned the entire length of the trachea down to the carina (Figure 3). There is associated luminal narrowing, with its narrowest transverse diameter measuring 0.7 cm. These findings were consistent with tracheobronchopathia osteochondroplastica (TBPO). There was incidental note of reticulonodular infiltrates in the posterior segment of the right upper lobe, lateral segment of the middle lobe, and superior segment of the right lower lobe, which are suspicious for tuberculous infiltrates given its endemicity in the Philippines. Thyroid function tests were unremarkable. Neck ultrasound revealed an enlarged right thyroid lobe and bilateral thyroid nodules. A fine needle aspiration biopsy of the most suspicious nodules was also done and proved to be benign.

**Figure 4.**
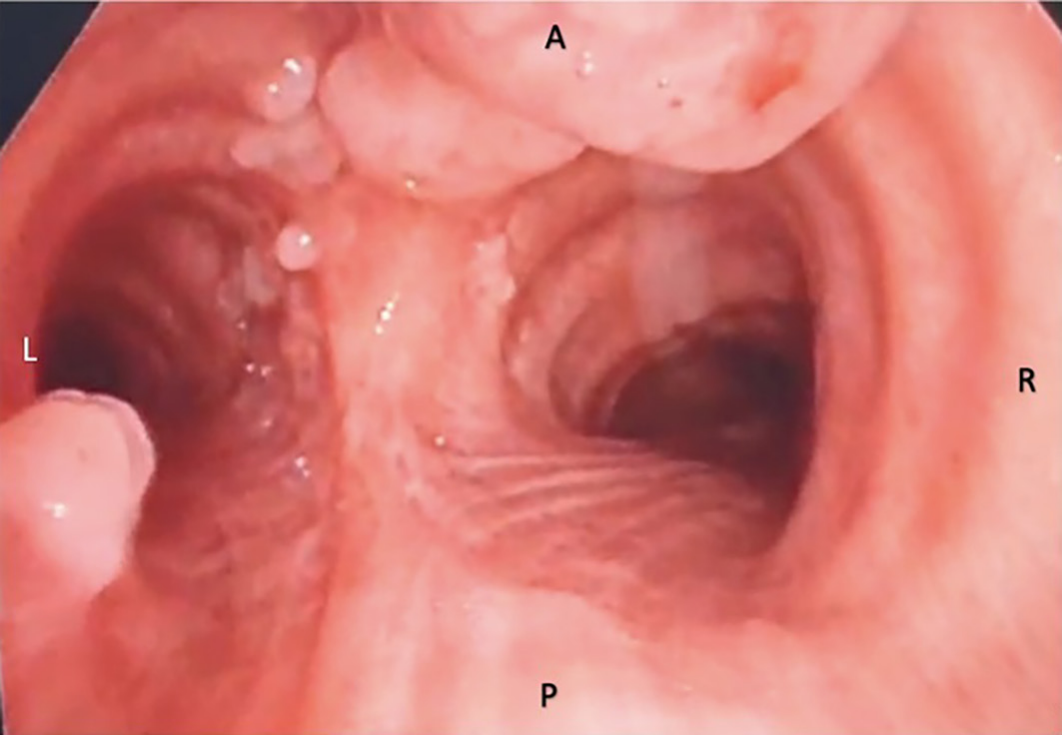
Wart-like lesions along anterolateral aspect of tracheal walls seen down to the level of the bifurcation of the carina.

Direct laryngoscopy and fiberoptic bronchoscopy were then performed to further characterize the extent of the lesions and obtain specimens for histopathological confirmation. These procedures again showed the wart-like lesions along the anterolateral tracheal walls beginning at the level of the subglottis down to the level of the carina (Figure 4). No further lesions were identified in the bronchopulmonary segments. Biopsy of the subglottic masses was done revealing fragments of mature bone tissue. Bronchial washings were negative for acid fast bacilli and mycobacterium tuberculosis was not detected on RT-PCR.

## Differential diagnosis

In the presence of multifocal nodules along the tracheal walls, the common differentials include amyloidosis, sarcoidosis, polychondritis, and papillomatosis; however, these disease entities involve the posterior wall.^1,2^

In our patient, the use of CT scan was necessary to suggest the diagnosis as the appearance of the lesions on flexible laryngoscopy may be similar to the lesions of recurrent respiratory papillomatosis. The rarity of this disease process makes it difficult to be recognized in practice.

## Treatment

Medical treatments are usually employed for symptomatic relief, since complete removal of all the nodules cannot be done.^3^ Once the patient was educated about the cause of her symptoms, no treatment was opted.

## Outcome and Follow-Up

The patient was instructed to follow-up at the outpatient clinic for progression/worsening of symptoms.

## Discussion

TBPO is a rare disease in which the presence of osseous and/or cartilaginous submucosal nodules projecting into the anterior and lateral walls of the airways is characteristic. There is sparing of the posterior membranous wall since the nodules originate from the cartilaginous rings.^4^ To the knowledge of the authors of this paper, there have been limited reports of this disease entity in the Philippines.

There have been several theories regarding the pathogenesis of this disease, however, no consensus has been reached. The two most common theories proposed include the development of ecchondrosis and exostosis by Virchow in 1869, and metaplasia of elastic tissue by Aschoff in 1910. Recently, Tajima et al. (1997) has discovered bone morphogenetic protein-2 (BMP-2) and transforming growth factor beta-1 (TGF-β1) as factors that induce new bone formation.^5^

Clinical diagnosis of this disease is difficult, as it is often asymptomatic. However, if symptoms do occur, patients present with non-specific complaints such as chronic cough, hemoptysis, dyspnea, stridor, wheezing, recurrent pneumonia, pleuritic chest pain, and foreign body sensation.^3,6^ Bronchoscopy is considered the gold standard in diagnosis by some authors with descriptions of a cobblestone, beaded, stalactitic cave, rock garden, or mountainscape appearance; however, its invasive nature can be a drawback.^7,8^ If the larynx and upper airway are involved, then visualization with laryngoscopy can be considered enough for diagnosis.^8^

CT scan can also be used to suggest the diagnosis, if pathognomonic findings of multiple calcified nodules along the anterior and lateral walls of the tracheobronchial tree without involvement of the posterior wall are seen, with irregularity of the tracheal morphology and decrease in the lateral diameter.^2,3,9^ A study by Zhu et al. (2014) in a population of 22 patients with TO had only a positive rate of 81.82% (*N* = 18/22), while a study by Luo et al. (2018) had a positive rate of 87.5% (*N* = 28/32).^2,10^

The need for histopathologic diagnosis is controversial, with some authors claiming that as the disease is asymptomatic with a benign course, biopsy is unnecessary, except when the pathognomonic appearance on imaging or bronchoscopy is not visualized.^3,9^^,^^11^

## Learning points

- Radiologic imaging is able to demonstrate the characteristic osseous nodules along the tracheal wall, with sparing of the posterior wall, thus allowing the radiologist to suggest the diagnosis of tracheobronchopathia osteochondroplastica.
- The extent of the lesions was also demonstrated accurately by the CT scan when compared to bronchoscopy, in this case.
- Although confirmation with laryngoscopy and bronchoscopy is standard procedure for evaluation, being able to characterize the lesions on CT scan is also as important in making the diagnosis.
- Familiarity with this rare disease is important in order to be able to recognize it in clinical practice.
- Communication between the radiologist and the clinician should be emphasized in order to properly clinch the diagnosis.
